# Effect of Oxidative Stress on Mitochondrial Damage and Repair in Heart Disease and Ischemic Events

**DOI:** 10.3390/ijms252212467

**Published:** 2024-11-20

**Authors:** Paweł Kowalczyk, Sebastian Krych, Karol Kramkowski, Agata Jęczmyk, Tomasz Hrapkowicz

**Affiliations:** 1 Department of Animal Nutrition, The Kielanowski Institute of Animal Physiology and Nutrition, Polish Academy of Sciences, Instytucka 3, 05-110 Jabłonna, Poland; 2 Student’s Scientific Association, Department of Cardiac, Vascular and Endovascular Surgery and Transplantology, Faculty of Medical Sciences in Zabrze, Medical University of Silesia, 40-055 Katowice, Poland; 3 Silesian Centre for Heart Diseases in Zabrze, Department of Cardiac, Vascular and Endovascular Surgery and Transplantology, Medical University of Silesia, 40-055 Katowice, Poland; thrapkowicz@sum.edu.pl; 4 Department of Physical Chemistry, Medical University of Bialystok, Kilińskiego 1, 15-089 Białystok, Poland; kkramk@wp.pl; 5 Students’ Scientific Association, III Department of Cardiology, School of Medical Sciences in Zabrze, Medical University of Silesia, 40-055 Katowice, Poland; aga.jeczmyk@gmail.com

**Keywords:** DNA exocyclic adducts, DNA repair, mitochondria damage, oxidative stress, hemostasis

## Abstract

The literature analysis conducted in this review discusses the latest achievements in the identification of cardiovascular damage induced by oxidative stress with secondary platelet mitochondrial dysfunction. Damage to the platelets of mitochondria as a result of their interactions with reactive oxygen species (ROS) and reactive nitrogen species (RNS) can lead to their numerous ischemic events associated with hypoxia or hyperoxia processes in the cell. Disturbances in redox reactions in the platelet mitochondrial membrane lead to the direct oxidation of cellular macromolecules, including nucleic acids (DNA base oxidation), membrane lipids (lipid peroxidation process) and cellular proteins (formation of reducing groups in repair proteins and amino acid peroxides). Oxidative changes in biomolecules inducing tissue damage leads to inflammation, initiating pathogenic processes associated with faster cell aging or their apoptosis. The consequence of damage to platelet mitochondria and their excessive activation is the induction of cardiovascular and neurodegenerative diseases (Parkinson’s and Alzheimer’s), as well as carbohydrate metabolism disorders (diabetes). The oxidation of mitochondrial DNA can lead to modifications in its bases, inducing the formation of exocyclic adducts of the ethano and propano type. As a consequence, it disrupts DNA repair processes and conduces to premature neoplastic transformation in critical genes such as the *p53* suppressor gene, which leads to the development of various types of tumors. The topic of new innovative methods and techniques for the analysis of oxidative stress in platelet mitochondria based on methods such as a nicking assay, oxygen consumption assay, Total Thrombus formation Analysis System (T-Tas), and continuous-flow left ventricular assist devices (CF-LVADs) was also discussed. They were put together into one scientific and research platform. This will enable the facilitation of faster diagnostics and the identification of platelet mitochondrial damage by clinicians and scientists in order to implement adequate therapeutic procedures and minimize the risk of the induction of cardiovascular diseases, including ischemic events correlated with them. A quantitative analysis of the processes of thrombus formation in cardiovascular diseases will provide an opportunity to select specific anticoagulant and thrombolytic drugs under conditions of preserved hemostasis.

## 1. Introduction

**Figure 1:**
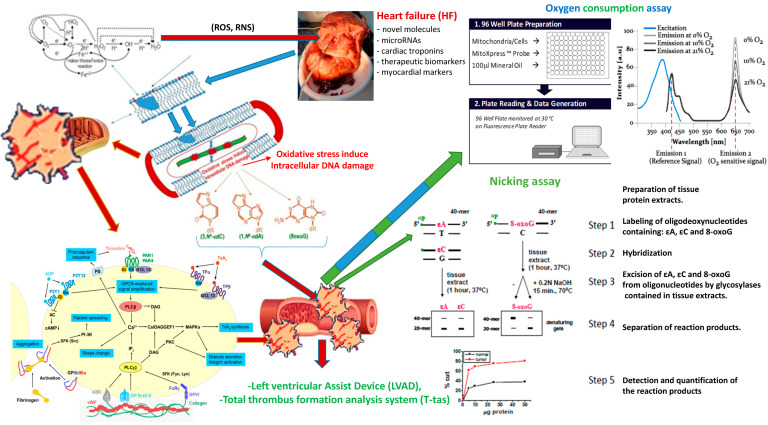
Methods related to platelet analysis (LVADs, T-Tas, nicking assay, and oxygen consumption assay) (own driving).

Heart diseases and ischemic events are the most common cause of death in the world [1,2,3]. The dominant cause is broadly defined heart failure (HF). Despite medical progress and numerous special programs targeted by European and American funds, the percentage of people dying each year reaches up to 30% in relation to other diseases in the cardiovascular category [1,2,3]. Heart failure (HF) is a complex and multifaceted syndrome that is associated with progressive multi-organ involvement, leading to the dysfunction of many systems in advanced stages [4,5]. It is characterized by a high mortality and morbidity rate [6]. Several pathways are involved in the pathophysiology of HF, which are key therapeutic targets. The development of molecular techniques has allowed for the identification of new molecules as potential biomarkers of diagnostic, prognostic, and therapeutic importance. In this case, microRNAs have been identified as promising tools, while other myocardial markers, such as neurohormonal natriuretic peptides or high-sensitivity cardiac troponins, have shown prognostic power, helping cardiologists in the management of patients with HF [5,7,8]. They may play a particular role in guiding pharmacological management and identifying the subclinical worsening of HF requiring therapy modification or more advanced treatment strategies [4,7,8] (Figure 1).

Among the numerous therapies used in heart diseases, the most common is mechanical circulatory support (MCS), which is becoming a standard therapy for patients with advanced HF with a significant impairment of ventricular ejection fraction [4,5,6,7,8,9]. MCS, in most cases, provides long-term support to the myocardium and is a bridge to heart transplantation (BTT) or a platform for myocardial remodeling/regeneration [9,10]. It is also often used as a destination therapy (DT) [11,12]. The number of patients receiving MCS therapy has increased fourfold in the last five years just like after the introduction of (CF-LVADs) [13]. Mechanical circulatory support with implantable, durable, continuous-flow left ventricular assist devices (CF-LVADs) is an established surgical treatment option for patients with advanced heart failure refractory to guideline-based therapy. Although the survival rates of patients supported by CF-LVAD therapy are 90% at 6 months and 85% at 12 months [14], there are numerous complications in the form of ischemic episodes, bleeding, strokes, infections (especially in the driveline), progressive right ventricular failure, and multi-organ failure [15,16,17,18], presenting as a significant clinical issue for the entire team of specialists. The annual report prepared by the Interagency Registry for Mechanically Assisted Circulatory Support (INTERMACS) showed that bleeding is the most common postoperative complication in patients with HF undergoing implantation of MCS devices in the form of CF-VADs [19,20,21]. Understanding the cause, course of pathways, and mechanisms of the normotonic saline bolus group (NSB) response associated with CF-LVADs is still a challenge and a priority for researchers worldwide. The literature data have indicated the important role of platelet glycoprotein Iba (GPIbα) ectodomain shedding in predicting the risk of non-surgical bleeding after CF-LVAD implantation [22,23]. Analyses also consider the occurrence of high shear stress within CF-VADs as the main cause of platelet receptors GPIbα, GPVI, and GPIIbIIIa ectodomain shedding [22,23,24,25]. These platelet receptors, glycoprotein, and thrombin are required for platelet aggregation and activation at sites of vascular injury [23,24,25].

Platelets play a key role in hemostasis and thrombosis, allowing us to hypothesize that their altered function and/or damage may lead to hemorrhagic complications in patients with HF supported by CF-LVADs. The literature data have shown a high incidence of platelet apoptosis in patients with CF-LVADs [21,22,23,24,25]. CF-LVADs, using a high-speed rotor to suck blood from the left ventricle and pump it through the graft to the aorta, cause nonphysiologically shear stresses [26,27]. Exposure to excessive LVAD ejection fraction containing multiple and partially damaged red blood cells and platelet increased shear stress may result not only in platelet activation but also in triggering processes leading to apoptosis by inducing endothelium depolarization of the mitochondrial transmembrane potential and exposure to phosphatidylserine [26,27,28,29,30].

The basic blood count includes leukocytes, platelets, erythrocytes, hematocrit, and hemoglobin. The full blood count is often enriched with a description of red blood cell parameters (MCV, MCH, MCHC) and platelet parameters (MPV, PDW, and pct), granulocytes, lymphocytes, eosinophils, and basophils. Its individual components, interacting with drugs or being subjected to the effects of molecular oxygen, may react differently in response to the existing stress factor, affecting, for example, blood viscosity [31,32]. Blood viscosity within a given shear stress depends on the hematocrit value, plasma viscosity, and the rheological properties of erythrocytes (erythrocyte elongation and aggregation), which constitute 40% of the morphological elements of total blood volume [31,32,33,34]. Shear stress affects G protein activation. G protein-coupled receptors (GPCRs) are the most numerous and highly diverse group of membrane proteins responsible for transmitting and mediating external signals and stimuli across the lipid bilayer to effector sites located within the cell [35]. As a result, they participate in the regulation of many physiological processes in multicellular organisms. The binding of extracellular ligands initiates the signal transduction cascade by inducing conformational changes in the receptor that promote the activation of the heterotrimeric GTP-binding protein (G protein) to GDP. In the inactive state, GDP is bound to the α subunit. During activation, GDP is released from the α subunit to bind to the GTP protein, and then the α-GTP complex dissociates from the β γ subunits [36,37].

It can be assumed that long-term exposure to shear stress in the CF-LVAD environment has an additive role in platelet damage and the associated increased risk of bleeding complications. This necessitates the investigation of the contribution of the relevant intrinsic and extrinsic oxidative stress-induced signaling pathways leading to platelet apoptosis in CF-LVAD patients in order to minimize bleeding complications [38,39].

## 2. Platelet Mitochondria Biogenesis

Platelets are the smallest non-nucleated (anucleate) blood cells that contain typical cellular organelles, including mitochondria, which enable them to exhibit an active metabolism [40]. Platelets have a highly organized cytoskeleton, specific secretory granules, and a unique system of membrane receptors that determine their high reactivity. Mitochondria not only participate in energy metabolism and ATP production in platelets but are also the main factors in platelet activation and apoptosis; both of these events are crucial for platelet function and lifespan [41]. Platelets are primarily responsible for maintaining normal hemostasis by preventing hemorrhage during vascular injury. Hemostasis is achieved by a careful balance of platelet interactions with vascular components, cytokine mediators, fibrinolytic agents, and plasma-clotting mechanisms. They help initiate a vascular response leading to vasoconstriction and the formation of a hemostatic plug (via adhesion, activation, and aggregation) [42,43,44]. The blood-clotting cascade is then initiated by clot expansion and a massive release of platelet contents. The released factors also help promote tissue repair and resolve the repair process. Mitochondria have several modes of mtDNA replication that differ significantly from the nuclear mode of replication, including the strand displacement mode (SDM), ribonucleotide-embedded lagging strand replication, and coupled leading and lagging strand synthesis [45,46]. The mtDNA copy number is considered an indirect measure of mitochondrial function, and its quantification in peripheral blood primarily reflects the mtDNA copy number in leukocytes and platelets. The epigenetic regulation of platelet mtDNA is of particular importance, as a higher platelet mtDNA methylation may be a potential biomarker of cardiovascular disease (CVD) [45,46]. Platelets are activated during the adhesive events of primary homeostasis and initiation of the blood-clotting cascades. Until recently, it was assumed that mitochondria played the only role in this process and was energetic. However, new studies have shown the contribution of several mitochondrial functions to platelet activation, such as mitochondrial permeability transition (MPT), increased ROS generation [47,48], and collapse of the mitochondrial membrane potential (ΔΨ m). Platelet activation is mediated by several agonists, namely collagen, thrombin, and ADP are involved in the regulation of hemostasis [47,49]. Adenosine diphosphate (ADP) is a platelet agonist that causes platelet shape change and aggregation, as well as the generation of thromboxane A2, another platelet agonist, through its effects on P2Y1, P2Y12, and P2X1 receptors [50,51,52,53,54,55,56,57,58,59]. The activity of these agonists is mediated by a common increase in intracellular calcium. Increased calcium levels have been found in mitochondria, which also correlate with ROS imbalance in mitochondria and activation of the MPT pore. Strong platelet activation characterized by a drastic increase in mitochondrial and cytosolic calcium also appears to initiate the collapse of the mitochondrial membrane potential (ΔΨ m) via a cyclophilin D (CypD)-dependent mechanism [60]. Mitochondrial activation pathways also contribute to the altered structure of platelet apoptosis. Oxidative stress-induced mitochondrial damage may induce apoptosis and Parkinson’s disease by reducing the levels of the oxidative protective protein, methionine sulfoxide reductase type 2 (Msrb2), and lactoferrin in the platelets of patients with Parkinson’s disease, leading to increased platelet apoptosis. Platelet mitochondria may serve as an important biomarker in neurodegenerative diseases that, either through genetic defects or environmental stress, lead to apoptosis and premature platelet death [61,62,63,64,65,66].

Increased mitochondrial maturation and differentiation (biogenesis) and oxidative stress in them under the influence of ROS lead to mitochondrial DNA damage and mutations due to external factors (ionizing radiation, radiation) or internal factors (lipid peroxidation, formation of modified DNA bases such as etheno or propano due to interactions of active substances of drugs). Interactions with substituents and heterocyclic rings which are very reactive and susceptible to the action of free radicals interacting with them form new oxidized hybrids such as enals, amides, etc. [67,68,69,70].

### 2.1. Genotoxic Properties of Endogenous Mitochondrial DNA Damage in Platelets

**Figure 2:**
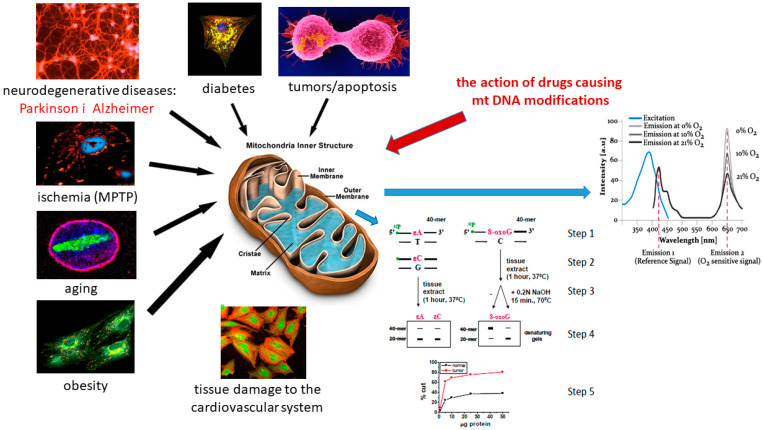
Principle of mitochondria dysfunction (own driving) and analyzed by methods of nicking assay and oxygen consumption assay [77,78].

Understanding the etheno DNA adducts, such as 1,N^6^-ethenoadenine (εA), 3,N^4^-ethenocytosine (εC), N^2^,3-ethenoguanine (εG), and 1,N^2^-ethenoguanine (εG), and repair pathways, resulting in the so-called oxidative stress process, is fundamental to understanding the mechanisms of diseases that depend on chronic inflammation such as cancer or neurodegenerative diseases induced by damages to mitochondria in platelets and aging processes. Due to the large range of these products and pleiotropic action, knowledge about the molecular mechanisms of their action is still fragmentary [71,72,73,74,75,76]. The principle of the method is based on cleavage of the oligodeoxynucleotide at the site of modified bases (exocyclic DNA base adducts) such as ethenoadenine (εA), ethenocytosine (εC), and ethenoguanine (εG) by glycosylases and AP-endonucleases contained in tissue homogenates (Figure 2). Due to the large range of these products and pleiotropic action, knowledge about the molecular mechanisms of their action is still fragmentary. It was assumed that the factor determining the course of the reaction is the amount of glycosylase, so DNA cleavage by glycosylases was determined by cleavage of the oligodeoxynucleotide [77,78].

### 2.2. Disturbance of Mitochondrial Function in Platelets

The disturbance of mitochondrial function and thus platelet function leads to their excessive activation and formation of a thrombus inside arteries and veins, which can lead to stroke. Interestingly, thrombosis of epicardial coronary arteries in ACS has been described in countless articles, whereas pathological changes in epicardial coronary veins have remained almost unnoticed. To our knowledge, there are several studies in the literature assessing the incidence of venous thrombosis in ACS [79,80,81,82,83,84,85].

Platelets, as one of the essential morphological components of blood, play a particularly important role in the development of acute coronary syndromes (ACSs) and in thromboembolic complications during and after percutaneous coronary intervention (PCI) [86]. It therefore seems important to review contemporary antiplatelet drugs in terms of their usefulness in the treatment of various forms of acute coronary syndromes. Acute coronary syndrome (ACS) is a particularly important cardiovascular complication in patients with cancer [87]. The occurrence of an acute thrombotic event in patients with cancer is associated with significant morbidity and mortality. As the prevalence of cardiovascular risk factors increases in aging cancer patients who survive longer, questions regarding the appropriate treatment of vascular toxicity with appropriate drugs are likely to become even more important in the years to come. The principles of use of these drugs, the limitations associated with them, and above all, the phenomenon of resistance are currently the subject of very intensive experimental and clinical studies. Antiplatelet drugs currently used in interventional treatment and chronic therapy in patients with ACS include acetylsalicylic acid, thienopyridines (clopidogrel and ticlopidine), and platelet glycoprotein receptor IIb/IIIa blockers (abciximab, eptifibatide, and tirofiban). Each of these drugs (or groups of drugs) blocks a specific pathway of platelet aggregation as follows: acetylsalicylic acid are dependent on thromboxane A2; thienopyridine on ADP; and GPIIb/IIIa receptor blockers are a pathway dependent on the fibrinogen receptor and other drug ingredients [88,89,90,91] also obtained from research on specific strains of bacteria. The European Society of Cardiology recommends acetylsalicylic acid and clopidogrel in the treatment of all patients with ACS, while GPIIb/IIIa receptor blockers are primarily used before and during procedures [92,93,94,95,96,97].

It has been unequivocally proven that antiplatelet drugs reduce the frequency of new adverse cardiac events in patients with acute coronary syndromes. However, their serious limitation is the phenomenon of resistance. It concerns practically all of the above-mentioned drugs and is associated, among others, with the occurrence of stent thrombosis, the consequence of which is death in 45% of cases [92,93,94,95,96,97]. Therefore, based on the available methods of a nicking assay, oxygen consumption assay, LVADs, and T-Tas (Total Thrombus formation Analysis System) an automated microchip flow chamber system for the quantitative analysis of the thrombus formation process under variable flow conditions [97,98,99,100,101] is necessary to develop standard, rapid, and reliable tests that will help identify patients resistant to individual antiplatelet drugs, which will contribute to preventing adverse cardiovascular events [9,10,13,19,21,97,98,99,100,101].

Autopsy revealed epicardial coronary venous thrombosis in 16 of 50 cases of left ventricular ACS. Furthermore, all venous thrombi were located in the veins of the infarcted myocardium. The vast majority of all myocardial infarctions (MIs) are caused by plaque rupture followed by coronary artery occlusion. However, a proportion of MIs, ranging from 1% to 14%, occur in the absence of coronary artery obstruction [102,103,104]. This phenomenon has been defined as myocardial infarction without significant coronary artery obstruction and has been the subject of extensive cardiovascular research. There are various etiological factors that cause significant coronary artery obstruction, such as myocarditis, microvascular disease, Takotsubo disease, and others [105]. All of these conditions can result in symptoms suggestive of myocardial ischemia and ST segment elevation. Surprisingly, the medical literature has paid a little attention to coronary venous thrombosis as a possible pathophysiological mechanism of significant coronary artery obstruction. Virchow’s triad includes three factors contributing to the development of thrombosis, which are venous congestion, vascular damage, and coagulation disorders. Venous congestion is the most important of the three factors, although congestion alone seems to be insufficient to cause thrombus formation [106]. However, the coexistence of venous congestion and vascular damage or coagulation disorders significantly increases the risk of thrombus formation. The clinical conditions most associated with venous thrombosis are generally related to the elements of Virchow’s triad [106,107,108,109]. These include surgery or trauma, malignancy, prolonged immobilization, pregnancy, congestive heart failure, obesity, advanced age, and a history of venous thrombosis [110,111,112,113,114,115,116]. These conditions have also been observed in coronary venous thrombosis.

Coronary sinus thrombosis is a rare complication usually associated with invasive procedures such as pacing lead implantation, central venous catheters, and ventriculoarterial shunts. It was reported that it can be a complication of fungal endocarditis, heart transplantation, mitral valve replacement, and heroin addiction [106,107,108,109,110,111,112,113,114,115,116]. Like thrombosis in other sites, such as cerebral, renal, mesenteric, or hepatic vein thrombosis, coronary venous thrombosis may be exacerbated by coagulation abnormalities, including factor V Leiden mutations, prothrombin gene mutations, antithrombin III deficiency, and protein C or protein S deficiencies. Only one report has shown that epicardial coronary venous thrombosis occurs in 32% of patients with MI [18]. Autopsies were performed on 63 patients with clinically definite or possible acute myocardial infarction (AMI) [116,117]. AMI was confirmed in 50 patients, and only this group underwent further pathological examination. Epicardial coronary vein thrombosis was demonstrated in 16 of 50 cases. Valve stenosis (aortic and mitral) was present in 6 of 50 patients, and venous thrombosis was demonstrated in all six cases [118,119,120,121].

### 2.3. Platelet Mitochondrial Metabolism Alterations

The main cause of the disruption of mitochondrial platelet metabolism is hypoxia or hyperoxia. Abnormal reactions in the Krebs cycle block the efficient generation of ATP. One of the effects of the above changes is platelet apoptosis. This can lead to their abnormal apoptosis, which contributes to the development of cardiovascular diseases, diabetes, and sepsis. In physiological conditions, cellular DNA and the nucleobases are constantly exposed to ROS, which causes their oxidative damage. This results in the formation of various forms of oxidized nucleotides, such as 8-oxo-2′-deoxyguanosine-5′-triphosphate (8-oxo-dGTP), 8-oxo-2′-deoxyadenosine-5′-triphosphate (8-oxo-dATP), 2-hydroxy-2′-deoxyadenosine-5′-triphosphate (2-OH-dATP), and 2-hydroxyadenosine-5′-triphosphate (2-OH-ATP) [13,14,15,16]. During the processes of DNA replication and transcription, oxidized nucleotides can be incorporated into DNA and induce incorrect base pairing during DNA replication, causing transversions. The end result of the described pathophysiological processes are genome instabilities and mutations [122,123,124,125,126,127,128,129,130].

## 3. Formation of Exocyclic Base Adducts in Platelet Mitochondrial DNA Under the Influence of Free Radicals Inducing Oxidative Stress and Lipid Peroxidation

The mitochondrial DNA of platelets are continuously exposed to a variety of external (drugs) and internal factors (lipid peroxidation) that alter its structure. These agents are both endogenous and exogenous and include normal cellular metabolism, cell injury, inflammation, ionizing radiation, and chemical agents. Analyzed data indicate that water, oxygen, and endogenous alkylation are the main contributors to overall DNA damage [70,71,72,73,74,75,76,77,124,126,129,131] creating exocyclic DNA adducts.

Of the exocyclic DNA adducts, etheno (ɛ) bases have been the most widely studied over the last 25 years, as they are formed by many genotoxic carcinogens, e.g., vinyl chloride or chloroacetaldehyde [70,71,72,73,74,75,76,77,124,126,129,131] and are also produced endogenously in animals and humans. This class of DNA lesions affects normal Watson–Crick base pairing in DNA and was shown to be mutagenic in *E. coli* and mammalian blood cells [70,71,72,73,74,75,76,77].

**Figure 3:**
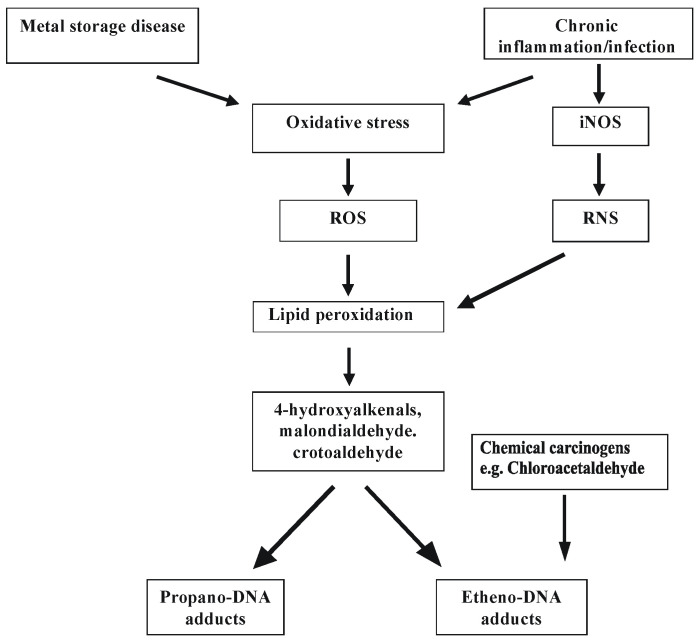
Proposed scheme of carcinogenic factors leading to oxidative stress-induced reactive oxygen species (ROS) and nitrogen (RNS) species; these can trigger lipid peroxidation that yields dialdehydes and alkenals, which cause exocyclic DNA base damage. PUFA, polyunsaturated fatty acid; iNOS, inducible nitric oxide synthase [135].

Etheno bases were first described by Kochetkov [127], who identified them as fluorescent analogs for biochemical studies and probes for nucleic acid structures [127], although among different exocyclic adducts, only 1,N^6^-ethenoadenine possesses fluorescent properties. The renewed interest in exocyclic DNA lesions in the 1990s was due to the development of ultrasensitive detection methods [46], notably for etheno and propano DNA adducts, which made it possible to study the formation of exocyclic adducts in experimental animals and humans. In 1994, the unequivocal identification of the malondialdehyde-derived deoxyguanosine (M1-dG) adduct was reported by Chaudhary et al. [130] in human liver. The same adduct was later also found in human breast and leukocytes by Vaca et al. [132]. In the years 1992–1999, Swenberg and co-workers [133,134,135] found background levels of etheno and propano adducts in DNA of various human and rodent tissues and confirmed the presence of N^2^,3-εdG in human liver by mass spectrometric techniques. These findings suggested an endogenous pathway for the formation of exocyclic adducts via lipid peroxidation products (Figure 3).

DNA adducts are mutagenic, teratogenic, and clastogenic in mammalian cells, to which they belong to etheno DNA bases such as ɛA, ɛC, and ɛG [70,71,72,73,74,75,76,77,124,126,129,131]. The genotoxic properties of all endogenous DNA damage were induced in processes called oxidative stress. The oxidative stress is the disturbances between all repair pathways, systems in the organisms, and their homeostasis [70,71,72,73,74,75,76]. Due to the large range of these products and pleiotropic action, knowledge about the molecular mechanisms of their action is still limited in the professional scientific literature. Understanding the genotoxic properties of endogenous DNA damage as etheno DNA adducts, such as 1,N^6^-ethenoadenine (εA), 3,N^4^-etheno-cytosine (εC), N^2^,3-ethenoguanine (εG), and 1,N^2^-ethenoguanine (εG) and repair pathways, resulting in the so-called oxidative stress process, is fundamental to understanding the mechanisms of diseases that depend on chronic inflammation such as cancer or neurodegenerative diseases and aging processes [70,71,72,73,74,75,76,77,124,126,129,131].

**Figure 4:**
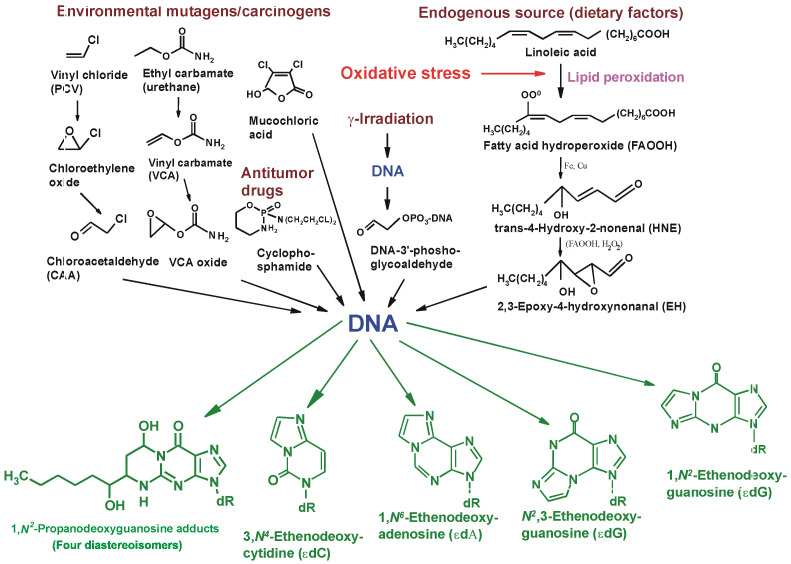
Major pathways for formation of exocyclic propano and etheno DNA adducts resulting from lipid peroxidation products and environmental mutagens/carcinogens [143] with modifications.

In the tissues of people potentially unexposed to teratogenic or mutagenic agents that promote the formation of DNA adducts, several exocyclic adducts have been found and quantified. These include 1,N^2^-propanodeoxyguanine (PdG) and etheno DNA adducts, such as 1,N^6^-ethenoadenine (1,N^6^-εA), 3,N^4^-ethenocytosine (3,N^4^-εC), N^2^,3-ethenoguanine (N^2^,3-εG), and 1,N^2^-ethenoguanine (1,N^2^-εG). It has been postulated that these lesions are formed in mammalian tissues under conditions of lipid peroxidation. The precise mechanism of formation of these lesions is unknown, although the formation of etheno adducts was observed in vitro when deoxyguanosine was exposed to 2,3-epoxy-4-hydroxynonenal (EH) [136,137]. Hydroxynonenal was shown in vitro to bind to deoxyguanosine and form a 1,N^2^-propano adduct with a hexyl side chain (Figure 4). These adducts were found in rodent and human DNA in the range of 1.8–15.8 adducts/10^8^ nucleotides [138,139,140,141,142] (Figure 4).

In all analyzed organisms (bacteria and mammals), etheno DNA adducts induce different types of mutations such as base substitutions, frameshift mutations, and sister chromatid exchanges and chromosomal aberrations. Etheno DNA may induce the inhibition of DNA synthesis on both strands in all types of the cells. However, replicative DNA polymerases such as alpha, kappa, or beta tend to incorporate non-cognate nucleotides opposite etheno adducts. This may lead to specific cancer-prone mutations with a high frequency, which are dependent on the source and type of DNA polymerase [70,71,72,73,74,75,76,77,138,139].

Studies of site-directed mutagenesis revealed that in bacteria, ɛA is often recognized as adenine by all synthesis DNA polymerases. It is also recognized sometimes infrequently, at about 0.1%, in the SOS mechanism, where the mechanism decreased replication fidelity caused by the production of very specific damaged DNA by polymerases of the Y family, which can induce AT→TA transitions [143] and AT→GC, AT→CG transversions and big fragments of nucleotide deletions. To compare, in simian kidney COS cells, 70% of eA residues in DNA are replicated erroneously, with the most frequent mutation being the AT→GC transition [59]. The literature data show that the mutagenic specificity of ɛA may strongly depend on its position on leading or lagging strands during DNA replication. For example, in human HeLa cells, AT→TA transversions were the most frequent, at 7% on the leading strand, although AT→CG and AT→GC base pair substitutions were also visible, at 5 and 2%, respectively [140,141,142,143,144,145,146], (Table 1).

Although both prokaryotic and eukaryotic cells are equipped with diverse DNA repair systems [70,71,72,73,74,75,76,77,140,141,142,143,144,145,146], the removal of DNA lesions in an error-free way sometimes is not efficient enough and damage escapes processing before replication. Unrepaired DNA damage leads to various biological consequences, such as mutations or cell death, subsequently to carcinogenesis, aging, and degenerative diseases [70,71,72,73,74,75,76,77,140,141,142,143,144,145,146].

It has been estimated that chronic inflammation in blood is involved in the development of about one-fourth of all cancers worldwide [140,141,142,143,144,145,146]. Inflammatory response leads to the recruitment of activated leukocytes, which release high quantities of reactive oxygen species (ROS) such as superoxide and hydrogen peroxide. Hydrogen peroxide can produce hydroxyl radicals in reaction with metal ions. Direct proof comes from the work of Dizdaroglu et al. [146], who showed that exposure of human cells to activated leukocytes causes DNA base modifications typical of a hydroxyl radical attack. ROS also interact with membrane lipids, causing their fragmentation and the production of reactive aldehydes, which are able to interact with nucleic acids and form exocyclic DNA adducts. These compounds can induce inflammation to pre-cancerous changes in tissues [70,71,72,73,74,75,76,77,146].

## 4. The Effect of Molecular Oxygen on Platelet Mitochondria as a New Marker of Oxidative Stress Measured by the Oxygen Consumption Assay Method

Molecular oxygen is a key substrate for aerobic metabolism, specified to monitoring cell oxidation, mitochondrial function, and metabolic implications of cell signaling. Its presence in tissues enables the real-time evaluation of transient changes in cellular respiration, oxygen gradients, and physiological responses in various cell models. One of the methods to measure the level of intracellular oxygen is the method that allows for the analysis of the concentration of molecular oxygen in a single layer of cells and their transient changes in metabolic activity in real time (oxygen consumption assay) [147] and T-Tas [97,98,99,100,101]. Understanding the factors controlling the release of HNO from its donors is a crucial issue when designing donors with specific properties. Now, for the first time, intracellular O_2_ can be conveniently monitored within the cell monolayer on a plate reader in a non-invasive, high-throughput manner and in real time. This is achieved using the new method known as the oxygen consumption assay by using the MitoXpress Xtra set [147]. This probe is an oxygen-sensitive phosphorescent metalloporphyrin based on the ability of O_2_ to quench the excited state of the assay. Because the concentration of oxygen within the cell monolayer can be depleted, this depletion is seen as an increase in probe signal intensity, expressed as real-time phosphorescence. The MitoXpress Xtra probe provides a powerful tool for the detailed investigation of this most critical of biological parameters like the analysis the concentration of molecular oxygen within the cell monolayer; a real-time assessment of transient changes in cell respiration, concentration, and consumption; hypoxia and hyperoxia processes; mitochondrial platelet function; and the metabolic implications of cell signaling, oxygen gradients, and physiological responses across a range of cell models [147]. So far, no research results have been published in the professional literature that the application of both methods would answer these questions. The oxygen consumption assay method using the MitoXpress Xtra test makes it possible to measure the complex dynamics of the intracellular oxygen metabolism of monolayers in hypoxic conditions [147]. MitoXpress-Intra probe is excitable at 340–400 nm and emissions are collected between 640 nm and 660 nm. Phosphorescent intensities are measured at delay times of 30 μs and 70 μs (with a 30 μs window time) with the ratio of these intensities subsequently converted to phosphorescent lifetimes. Extracellular oxygen consumption by cells was measured on a multi-mode microplate filter reader (FLUOstar OPTIMA, BMG Labtech, Offenburg, Germany). In this assay, MitoXpress Xtra is quenched by O_2_ through molecular collision; thus, the magnitude of the fluorescence signal is inversely proportional to the amount of extracellular O_2_ in the sample. Rates of oxygen consumption are calculated from the changes in the fluorescence signal over time. The reaction is not destructive and fully reversible (neither MitoXpress Xtra nor O_2_ are consumed), facilitating the measurement of time courses and antiplatelet, antithrombotic, and thrombolytic drug treatments [147].

The T-TAS^®^ system developed by the Japanese company Fujimori Kogyo is a system for the comprehensive and quantitative analysis of platelet plugs or thrombus formation under semi-physiological conditions in blood flow through artificial blood vessels (microcapillaries) placed in tests. The microcapillaries are coated with collagen (PL test) or collagen and tissue thromboplastin (AR test or HD test), respectively. It is extremely useful in the study of hypoxia contained in platelet mitochondria under the inflammation of various pharmacological compounds. These analyses are currently widely used in many areas of cell biology (including cell differentiation and proliferation, intercellular interaction, cancer, and toxicology). The use of drugs that activate or reduce the action of platelets and various environmental compounds can lead to the action of platelet mitochondria by excessive delivery or depletion of molecular oxygen [97,98,99,100,101].

Platelet mitochondria are a new key marker in the analysis of oxidative stress in living organisms as a new marker of oxidative stress in ischemic heart diseases with interventional medicine [148,149,150].

Chronic inflammatory infection is one of the sources of free oxygen radicals. It also leads to nitric oxide synthase (NOS) induction and therefore to NO synthesis. Oxidative stress processes mainly interact inside the cell and enhance the generation of reactive oxygen species such as O_2_, H_2_O_2_, and OH. The most reactive molecule is the hydroxyl radical. Its production can be increased in response to the accumulation of free Cu and Fe ions in tissues (mainly in the liver), which is known to occur in some procancerogenic diseases, Wilson disease, and primary hemochromatosis. These transient metal ions participate in Fenton and Haber–Weiss reactions to produce hydroxyl radicals according to [151,152,153].

Fe^2+^ + H_2_O_2_ → OH + OH^−^ + Fe^3+^

O_2_^·−^ + Fe^3+^ → O_2_ + Fe^2+^

Haber–Weiss reactions:

Fe^2+^/Fe^3+^

O_2_^·−^ + H_2_O_2_ → OH^•^ + OH^−^ + O_2_

**Figure 5:**
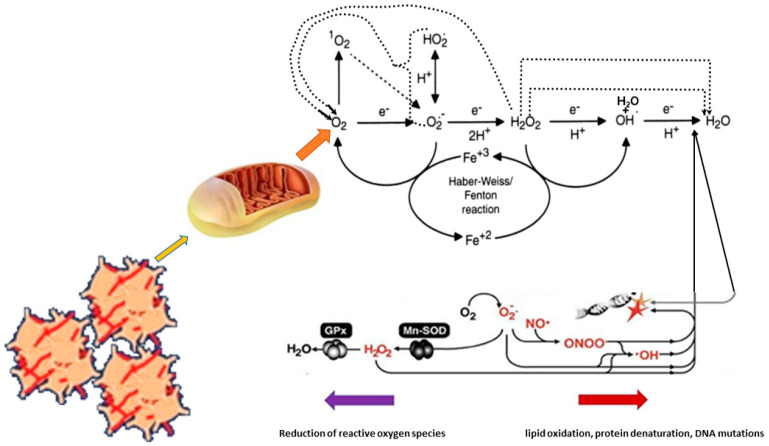
Oxidative changes inside the mitochondria of blood platelets. https://biologydictionary.net/mitochondria/, accessed on 1 January 2020 with own modifications.

Myocardial cellular ROS are generated endogenously as byproducts of mitochondrial oxidative phosphorylation or as intermediates of oxidoreductase enzymes and metal-catalyzed oxidation. Because oxygen atoms contain two unpaired electrons in separate orbitals of the outer electron shell, they are susceptible to the formation of radicals. The sequential reduction of oxygen by the addition of electrons leads to the formation of a range of ROS, including superoxide anions (O_2_^·−^), hydrogen peroxide (H_2_O_2_), hydroxyl radicals (^•^OH), hypochlorous acid (HOCl), peroxynitrite anions (ONOO^−^), and nitric oxide (NO). Because of its deleterious effects, cells have several carefully regulated systems for managing excess ROS [154,155]. The best-known system is the glutathione–ascorbate cycle, which detoxifies H_2_O_2_ to H_2_O using NADH and NADPH as electron donors. Other systems include enzymes such as superoxide dismutase, which catalyzes the dismutation of superoxide anions (O_2_^·−^) to O_2_ or H_2_O_2_, and catalase, which catalyzes the decomposition of H_2_O_2_ into H_2_O and O_2_. The detection of the intracellular hydroxyl radical (^•^OH) is crucial for understanding normal cellular redox regulation and the impact of its dysregulation on various pathologies [153,154,155]. The hydroxyl radical is one of the reactive oxygen species (ROS) that are highly reactive with other molecules to achieve stability. In general, hydroxyl radicals are considered to be a harmful byproduct of oxidative metabolism that can cause molecular damage in living systems. It exhibits an average lifetime of 10^−9^ nanoseconds and can react with almost any biomolecule, such as nuclear DNA, mitochondrial DNA, proteins, and membrane lipids [155,156,157,158,159]. Sodium ions are negatively charged and therefore cannot directly cross the hydrophobic lipid bilayer of the cardiac cardiomyocyte cell membrane by themselves. Instead, they must use unique channel proteins known as sodium channels. In response to an increase in membrane potential to about −55 mV (in this case caused by an action potential), activation gates open, allowing positively charged Na+ ions to flow into the cardiomyocyte through the channels and causing the voltage across the neuronal membrane to increase to +30 mV in human neurons, leading to dysfunction caused by oxidative stress [155,156,157,158,159,160,161,162,163] (Figure 5).

## 5. Factors Inducing Excessive ROS Production in Cardiomyocytes as a Result of Platelet Mitochondrial Dysfunction

The heart muscle in healthy conditions works continuously throughout the entire human life. This is associated with a significant energy accumulation, in which the dominant metabolic process is aerobic respiration, which is responsible for 95% of the production of total ATP in the heart muscle. Heart muscle cells, cardiomyocytes, show a significantly higher concentration of mitochondria in relation to other types of cells [164,165,166]. Intensive metabolic changes occurring in mitochondria related to cellular respiration promote destabilization and rapid changes occurring in cells under the influence of various environmental factors. This leads to excessive and uncontrolled production of free oxygen radicals (ROS). Additionally, K^+^ and Ca^2+^ ion channel dysregulation results in improper ion flow, mitochondrial depolarization, and disturbed energy metabolism. The main mechanism responsible for cellular damage to cardiomyocytes and the induction of inflammatory processes creating a cascade of pathophysiological changes is the dysregulation of the ROS production hemostasis [164,165,166]. Each time such a disturbance of cellular homeostasis in the heart muscle occurs, it results in insufficient energy metabolism and cellular damage to cardiomyocytes [164,165,166].

The most common etiological factors contributing to the development of HF include arterial hypertension, diabetes, and obesity. Each occurrence of HF is associated with a significant disturbance of metabolic processes, insufficient energy supply in the myocardium, or abnormal redox processes that maintain the ROS balance under physiological conditions [165,166,167,168,169]. Disturbances in energy metabolism and insufficient energy supply are currently considered to be one of the factors significantly affecting mitochondrial damage. During myocardial remodeling, there is a decrease in the concentration of carnitine, a cofactor involved in the incorporation of fatty acids into metabolic processes in the beta-oxidation pathway. The decrease in the efficiency of fatty acid reduction results in a gradual change in the main energy pathway of the myocardium to glycolysis. However, glycolysis provides only 5% of the energy demand of the myocardium. An insufficient supply of ATP from glycolysis processes and lack of appropriate compensation from the fatty acid pathway leads to a gradual loss of ATP reserves [165,166,167,168,169,170]. Disruptions in the expression of MT-ND1, MT-ND5, and MT-ND6 genes responsible for the production of NADH phosphate (NADPH)-trans-hydrogenase result in a decrease in the concentration of NAD+ forms. This eliminates the protective function of NAD+ against oxidative stress and the overproduction of ROS [165]. In addition, the decrease in NAD+ concentration may also result from excessive substrate consumption, e.g., during inadequate metabolic processes [165]. Inducing processes related to lipid peroxidation, in which DNA bases are modified and etheno and propano derivatives are formed leads to a disturbance in carbohydrate metabolism in the body [70,71,72,73,74,75,76,77,165].

Diabetes and carbohydrate metabolism disorders are another factor inducing oxidative stress. Hyperglycemia, through the glycation of metabolic products, including proteins and mitochondrial DNA, results in the formation of pathological connections that structurally damage mitochondria. The induction of mitochondrial oxidative stress destabilizes the redox processes occurring, disrupting the integrity of mitochondrial membranes. Hyperglycemia and insulin resistance disrupt the processes of ATP synthesis through electron transport through the mitochondrial chain. The inefficiency of the above process triggers significant ROS production in order to avoid mitochondrial damage by significant amounts of free electrons. This negatively affects the electron transport chain itself and, ultimately, energy metabolism. In addition, oxidative damage to proteins, lipids, DNA, and other mitochondrial structures occurs [165].

Cellular interactions initiated through multiple signaling pathways have a significant impact on ROS production within mitochondria. One of the most important is a signaling pathway involving Toll-like receptors (TLRs). In the context of mitochondrial disorders, we are talking about the receptor-mediated activation of TLR1, TLR2, and TLR4 subtypes. Factors binding to the mentioned subtypes include lipopeptides, mannans, hemagglutinin protein, heat-shock protein, fibrinogen, and others [165].

Essential in the perspective of mitochondrial oxidative stress is the activation of TLR2. Frequently induced by Gram-positive bacteria, it stimulates the production and secretion of NO, one of the reactive forms of ROS. The regulation of NO concentration is conducted with the participation of iNOS. Impaired Nrf2 activation has been demonstrated in patients with diagnosed coronary artery disease (CAD). This results an impairment of c-Jun-mediated signaling and *COX-2* overexpression. This leads to the inhibition of *PI3K* and *NF-κB* and the disruption of *Nrf2*-mediated antioxidant reactions.

The above mechanism confirms the highly significant influence of the external environment on the induction of oxidative stress independent of organic changes [144,155,156,157,158,159,160,161,162,163,164,165].

The final effect of the synergistic action of the above processes is the induction of mitochondrial oxidative stress, which disrupts signaling pathways and the energy management of the cell and can lead to the apoptosis of many tumor suppressor genes, including oncogenes, e.g., the *p53* gene or, specifically, proteins like lactoferrine [171].

## 6. Fatty Acid Oxidation During Diabetes Disrupts the Energy Management of Platelet Mitochondria

Fatty acid oxidation during diabetes disrupts the energy management of mitochondria through the accumulation of fatty acids in cardiomyocytes, affecting the increase in the concentration of diacylglycerols and ceramides. Diacylglycerols, by activating C-kinases (PKCs), intensify cellular insulin resistance, and in a feedback mechanism, as well as in ROS production, the creation of oxidative stress, and inflammatory processes [171,172,173]. Disturbances of mitochondrial Ca^2+^ management are crucial for considering the role of mitochondria and oxidative stress in inducing cardiomyocyte damage. This results from the very dependence of transmembrane Ca^2+^ ion transport across the membranes of the endoplasmic reticulum. These processes, which require ATP-dependent transmembrane channels, are characterized by a significant energy demand [172]. Mitochondrial Ca^2+^ channels, by constantly balancing the endoplasmic ion concentration, constitute a specific ion buffer preventing Ca^2+^ deficiency or overload inside cardiomyocytes [173]. The disturbance of the delicate homeostasis in the transmembrane transport of Ca^2+^ ions leads to a pathological activation of other mitochondrial transmembrane channels, increasing the probability of electron leakage, ROS, and subsequent oxidative stress. The disturbed cytoplasmic Ca^2+^ concentration, at least by influencing the transport of other ions, including Na+, changes physiological depolarization processes, which can secondarily induce cardiac arrhythmias [174]. Additionally, indirectly, through an increase in Ca^2+^ concentration in vascular smooth muscle cells and an intensification of calcification processes, the myocardium is gradually overloaded. This forces the cardiomyocytes to work more intensively, thus increasing their energy demand. Insufficient mitochondrial metabolism is further destabilized [175]. The accompanying increase in Ca^2+^ concentration in cardiomyocytes and the recruitment of an increasing amount of mitochondrial calcium uniporter (MCU) intensify the dysfunction of energy metabolism processes. Cardiomyocytes, trying to compensate for the cytoplasmic excess of Ca^2+^, expand the membranes of the endoplasmic reticulum. This leads to pathological cell hypertrophy. Moreover, the increased number of MCU results in oxidative stress and an increase in ROS concentration, which in a feedback loop intensifies cellular damage [176].

Fe^2/3+^ ions play an important role in the regulation of many cellular pathways. The increase in cellular Fe^2/3+^ concentration that accompanies HF leads to gradual cell overload. This disrupts most of the key metabolic processes occurring in cardiomyocytes. The effect of long-term Fe ions is ferroptosis, leading to structural damage and thinning of the myocardium. The loss of functional cardiomyocytes impairs contractile function and overall cardiac efficiency [177]. Disturbances in the transport of metal ions, especially Fe and Mg^2+^, are currently considered as factors initiating ROS and oxidative stress. These ions actively participate in numerous metabolic pathways such as fatty acid oxidation, glycolysis, or in the electron transport chain. Non-physiological Fe ion overload increases ROS production while significantly disrupting energy metabolism occurring in the myocardium. Mg^2+^ ions via manganese-dependent superoxide dismutase (MnSOD/SOD2) and pyruvate carboxylase enzymes regulate mitochondrial ROS concentration. A decrease in Mg^2+^ concentration and enzymatic activity (MnSOD/SOD2) leads to an increase in mitochondrial ROS with simultaneous disruption of the functioning of enzymatic complex I of the mitochondrial transformation pathway [178].

## 7. The Influence of ROS-Induced Mitochondrial Dysfunction on Cardiovascular Damage

Oxidative stress via ROS through damage to platelet mitochondrial DNA and the associated dysfunction of mitochondria themselves can induce various types of diseases associated with ischemic incidents of the cardiovascular system, e.g., heart failure and modified myocardial cells (cardiomyocytes). Clusters of these cells form nodal tissue, the task of which is to generate and conduct electrical impulses that stimulate the heart to contract. They form a specific creative and conductive stimulus system of the heart [178].

ROS-induced heart failure is a chronic process that gradually leads to impaired cardiomyocyte contractile capacity and function. The typical picture is a disturbance of the contraction/relaxation phases, a decrease in the ejection fraction, remodeling of the organ itself and, ultimately, systemic circulatory system failure. The dysregulation of cardiomyocyte metabolic pathways by disrupting the processes of glycolysis and fatty acid oxidation prevents the proper functioning of the respiratory chain. This induces structural mitochondrial damage, increased oxidative stress, and enzymatic dysfunctions, including acyl-coenzyme A (CoA). A decrease in CoA concentration leads to cardiomyocyte hypertrophy [179,180]. In the case of hyperglycemia and diabetes, we are dealing with multifactorial processes. The overlap of disorders of the energy metabolism of fatty acids and Ca^2+^ management, loss of the protective function of the SOD and glutathione peroxidase 1 (GPX 1) genes, or overloading of cardiomyocytes with Fe^2/3+^ ions gradually impair their efficiency, leading to progressive failure. The accompanying structural disorders of mitochondria, apoptosis processes, and arrhythmias based on the improper depolarization of the myocardium through synergistic effects impair the overall efficiency of the heart muscle. Additionally, insulin resistance prevents the proper participation of insulin in metabolic signaling pathways [181,182,183,184,185,186,187,188,189].

## 8. Conclusions

Oxidative stress, ROS formation, and mitochondrial dysfunctions play a significant role in the pathophysiology of heart failure. Cardiomyocytes, as cells with significant energy requirements, require a constant balance of the changes occurring in them. Pathophysiological processes such as hypertension, diabetes, or acute conditions easily disturb homeostasis, leading to dysregulation at the mitochondrial level. Due to the redox processes occurring in them, this leads to the release of highly harmful ROS. This damages numerous cellular organelles and forces adaptation by inducing compensatory processes, ultimately leading to cardiomyocyte dysfunction. A contemporary understanding of the pathophysiology of HF will require an interdisciplinary approach considering both extracellular factors like α-amides and intracellular factors like bacterial pathogenic biofilms.

## Figures and Tables

**Table 1:** The types of base changes induced by etheno bases observed in vitro in *E. coli* and mammalian cells.

Lesion	Base Changes		
	In Vitro	*E. coli*	Mammalian Cells
εA	A→G, A→T > A→C	A→G > A→C, A→T	A→G > A→T, A→C
β	A→T > A→C	A→G, A→C, A→T	Not Determined
εC	C→A, C→T > C→G	C→T, C→A	C→A, C→T > C→G
εC^•^H_2_O	No Incorporation	C→T	Not Determined
N^2^,3εG	G→A	G→A	G→T, G→A
1,N^2^-εG	G→T, G→C	G→T, G→C, G→A	G→A > G→T
HO-ethanoG	G→T, G→C	G→T, G→C, G→A	Not Determined

## Data Availability

Data are provided upon request for those interested.

## Abbreviations

ROS	Reactive oxygen species
RNS	Reactive nitrogen species
mtDNA	Mitochondrial DNA
HF	Heart failure
MCS	Mechanical circulatory support
BTT	Bridge to heart transplantation
DT	Destination therapy
CF-LVAD	Mechanical circulatory support using implantable, durable, continuous-flow left ventricular assist device
INTERMACS	Interagency Registry for Mechanically Assisted Circulatory Support
NSB	Normotonic saline bolus group
GPIbα	Platelet receptor glycoprotein Ibα (GpIbα)
GPVI	Platelet receptor GPVI (glycoprotein VI)
GPIIbIIIa	Platelet glycoprotein IIb/IIIa receptors
MCV	Mean corpuscular volume
MCH	Mean corpuscular hemoglobin
MCHC	Mean corpuscular hemoglobin concentration
MPV	Mean platelet volume
PDW	Platel distribution width
Pct	Platelet hematocrit or thrombocrit parameter
GPCRs	G protein-coupled receptors
G protein	Guanine nucleotide-binding proteins
CVD	Coronary heart disease
MPT	Mitochondrial permeability transition
ADP	Adenosine diphosphate
P2Y1	Purinergic receptor
P2Y12	Oral platelet inhibitors
P2X1	Purinergic receptor
CypD	Cyclophilin D
Msrb2	Methionine sulfoxide reductase type 2
ΔΨ m	Mitochondrial membrane potential
(εA)	1,N^6^-ethenoadenine
(εC)	3,N^4^-ethenocytosine
(εG)	N^2^,3-ethenoguanine or 1,N2-ethenoguanine
ACS	Acute coronary syndrome
PCI	Percutaneous coronary intervention
ADP	Adenosine diphosphate
AMI	Acute myocardial infarction
(8-oxo-dGTP)	8-oxo-2′-deoxyguanosine-5′-triphosphate,
(8-oxo-dATP)	8-oxo-2′-deoxyadenosine-5′-triphosphate
(2-OH-dATP)	2-hydroxy-2′-deoxyadenosine-5′-triphosphate
(2-OH-ATP)	2-hydroxyadenosine-5′-triphosphate
M1-dG	Malondialdehyde-derived deoxyguanosine
(PdG)	1,N^2^-Propanodeoxyguanine
T-TAS	(Total Thrombus formation Analysis System) is an automated microchip flow chamber system for the quantitative analysis of the thrombus formation
MT-ND1, MT-ND5, MT-ND6	Genes responsible for the production of NADH phosphate (NADPH)-trans-hydrogenase
NADH	NADH phosphate
TLR1, TLR2 and TLR4	Receptor-mediated activation, mediates both pro- and anti-inflammatory responses
iNOS	Inducible nitric oxide synthase
Nrf2	Nuclear factor erythroid 2-related factor 2
c-Jun	Nuclear transcription factor
COX-2	Cyclooxygenase-2
PI3K	Phosphoinositide 3-kinase
NF-κB	Nuclear factor kappa-light-chain-enhancer of activated B cells
PKC	Protein kinase C
CoA	Coenzyme A
